# Accuracy of patient-specific I-131 dosimetry using hybrid whole-body planar-SPECT/CT I-123 and I-131 imaging

**DOI:** 10.1186/s40658-024-00657-9

**Published:** 2024-06-20

**Authors:** Michaella Morphis, Johan A. van Staden, Hanlie du Raan, Michael Ljungberg, Katarina Sjögreen Gleisner

**Affiliations:** 1 Department of Medical Physics, Faculty of Health Sciences, University of the Free State, Bloemfontein, South Africa; 2 Medical Radiation Physics, Lund University, Lund, Sweden

**Keywords:** Monte Carlo simulations, SIMIND, Hybrid whole-body planar-SPECT/CT, ^123^I, ^131^I, mIBG

## Abstract

**Purpose:**

This study aimed to assess the accuracy of patient-specific absorbed dose calculations for tumours and organs at risk in radiopharmaceutical therapy planning, utilizing hybrid planar-SPECT/CT imaging.

**Methods:**

Three Monte Carlo (MC) simulated digital patient phantoms were created, with time-activity data for mIBG labelled to I-123 (LEHR and ME collimators) and I-131 (HE collimator). The study assessed the accuracy of the mean absorbed doses for I-131-mIBG therapy treatment planning. Multiple planar whole-body (WB) images were simulated (between 1 to 72 h post-injection (p.i)). The geometric-mean image of the anterior and posterior WB images was calculated, with scatter and attenuation corrections applied. Time-activity curves were created for regions of interest over the liver and two tumours (diameters: 3.0 cm and 5.0 cm) in the WB images. A corresponding SPECT study was simulated at 24 h p.i and reconstructed using the OS-EM algorithm, incorporating scatter, attenuation, collimator-detector response, septal scatter and penetration corrections. MC voxel-based absorbed dose rate calculations used two image sets, (i) the activity distribution represented by the SPECT images and (ii) the activity distribution from the SPECT images distributed uniformly within the volume of interest. Mean absorbed doses were calculated considering photon and charged particle emissions, and beta emissions only. True absorbed doses were calculated by MC voxel-based dosimetry of the known activity distributions for reference.

**Results:**

Considering photon and charged particle emissions, mean absorbed dose accuracies across all three radionuclide-collimator combinations of 3.8 ± 5.5% and 0.1 ± 0.9% (liver), 5.2 ± 10.0% and 4.3 ± 1.7% (3.0 cm tumour) and 15.0 ± 5.8% and 2.6 ± 0.6% (5.0 cm tumour) were obtained for image set (i) and (ii) respectively. Considering charged particle emissions, accuracies of 2.7 ± 4.1% and 5.7 ± 0.7% (liver), 3.2 ± 10.2% and 9.1 ± 1.7% (3.0 cm tumour) and 13.6 ± 5.7% and 7.0 ± 0.6% (5.0 cm tumour) were obtained for image set (i) and (ii) respectively.

**Conclusion:**

The hybrid WB planar-SPECT/CT method proved accurate for I-131-mIBG dosimetry, suggesting its potential for personalized treatment planning.

## Background

Personalising cancer treatment in oncology is rapidly evolving, highlighting the importance and advantage of radiopharmaceutical therapy [1, 2]. Theragnostics is a field in nuclear medicine (NM) [3] where radiopharmaceuticals and imaging techniques are uniquely combined to sequentially diagnose and treat certain types of cancer.

Metaiodobenzylguanidine (mIBG) was first used to image tumours in the adrenal medulla as early as 1980. When labelled with iodine-123 (I-123) or iodine-131 (I-131), mIBG has become standard for the detection, staging and treatment of neuroendocrine tumours (NETs), including neuroblastomas, pheochromocytomas and paragangliomas in NM imaging [4, 5]. NETs have the potential to manifest in nearly any organ, with the gastrointestinal tract being their most prevalent location and the lung constituting the second most frequent primary site [6, 7] It is widely assumed that I-123 and I-131 labelled to mIBG demonstrate similar pharmacokinetic behaviour in patients with NETs, as both isotopes are labelled to the same pharmaceutical agent [8, 9]. Gear et al. [10] argued that I-123 presents potential uncertainties due to its short physical half-life compared to I-131. Consequently, the EANM dosimetry committee currently recommends the use of I-131 for pre-therapy dosimetry calculations [10]. Nevertheless, we presume that the pharmacokinetics are predicted similarly using either I-123 or I-131, acknowledging that some institutions may still prefer I-123 for pre-therapeutic dose calculation due to its superior image quality and reduced radiation exposure to both patients and staff.

I-123 (principal photon energy (159.0 keV)) can be imaged with either a low energy high resolution (LEHR) or a medium energy (ME) collimator. When an improved spatial resolution is required, the LEHR collimator is recommended; however, this is only accurate when appropriate corrections for septal scatter and penetration are applied since I-123 emits multiple high energy photons (e.g. 529 keV with 1.28% abundance) [11]. The ME collimator reduces the influence of septal penetration from the higher energy photons of I-123 and is thus the preferred collimator when—more accurate activity quantification is required. However, some NM clinics may only have access to a LEHR collimator; therefore, it is worthwhile investigating the LEHR collimators’ potential use for I-123 imaging. Due to the relatively high energy of the I-131 main photopeak (364.5 keV), together with the septal penetration and scatter from its’ higher energy photons (637.0 and 722.9 keV), the use of a high energy (HE) collimator is required for I-131 imaging [11–13]. In addition to I-131 gamma photons, beta particles with a maximum and mean energy of 606.3 keV and 191.6 keV are also emitted, making I-131 an ideal therapeutic agent.

Typically, the efficacy of cancer treatment is related to the high absorbed dose to the tumours. However, the absorbed dose limits for the organs at risk and the surrounding tissue must be considered [14]. Knowing the absorbed dose to tumours and organs at risk is important for ensuring the safety and optimisation of radiopharmaceutical therapy, especially when evaluating its radiobiological effect [15]. The effectiveness of radiopharmaceutical therapy can therefore be improved with accurate patient-specific dosimetry.

The mathematical formalism for internal dosimetry calculations has been described by the Medical Internal Radiation Dose Committee of the Society of Nuclear Medicine [16, 17], the Radiation Dose Assessment Resource and the International Commission on Radiological Protection, albeit with different nomenclatures [18]. In principle, the calculation of the absorbed dose to a given target region is based on determining two parameters: the time-integrated activity in various source regions and the radiation-energy transport from the source regions to the target region. Data on the second parameter has been tabulated as dose factors for standard reference geometries and radionuclides. However, absorbed dose calculations from standard geometries are, in principle, of limited use in radionuclide therapy with high absorbed doses. This is because organ shape, size, and position can vary considerably from patient to patient and personalized dosimetry calculation requires a more precise description of the patient than a standard mathematical phantom [19].

The use of quantitative SPECT/CT allows for an alternative to the tabulated dose factors, based on voxel-based Monte Carlo (MC) calculation. By treating each voxel of the SPECT image as a source region and using the CT image to estimate the probabilities for radiation interaction, the energy deposition in all other voxels (treated as target regions), including the voxel itself, can be calculated. The absorbed dose rate can thus be determined on a voxel-by-voxel basis. Absorbed dose rates to organs and tumours can then be quantified by proper image segmentation and averaging.

Patient-specific biokinetic information can be obtained from either planar (static or whole-body (WB)), SPECT/CT or a combination of planar and SPECT/CT images (referred to as the hybrid method) [20]. The drawback of using only planar imaging is its relatively poor image contrast due to over- and underlying activity distributions. Image contrast improves during SPECT imaging, allowing for more accurate activity quantification and dose calculations. However, a potential drawback of WB SPECT imaging for quantification purposes is the gamma camera detectors’ limited axial range. Performing SPECT imaging over several axial positions is considered impractical for most NM clinics with large patient load. By combining multiple WB planar images and a single SPECT/CT image, one can take advantage of the effectiveness and speed of WB planar imaging with the improved quantification accuracy of SPECT/CT imaging [21].

Undoubtedly, the accuracy of the hybrid method relies on the SPECT activity quantification accuracy. SPECT quantification accuracy depends on many factors; the camera system calibration, the radionuclide-collimator combination and the corrections applied for degrading factors incorporated into the reconstruction algorithm. These corrections include object scatter, non-homogenous attenuation, collimator detector response (CDR), collimator-septal scatter and septal penetration [20, 22].

Studies have shown that the hybrid imaging method for dosimetry outperforms planar-based methods, and its accuracy is comparable to SPECT/CT methods [23]. For the hybrid imaging method to be accurate, three factors should be considered prior to performing hybrid WB planar-SPECT/CT quantification of patients: (i) there should be minimal overlap between source and target regions with high activity uptake in the WB planar images, (ii) the volume, represented by the region of interest (ROI) on the WB image, should be included in the SPECT/CT field-of-view, and (iii) early and late uptake in the ROI should be clearly visible on the WB planar image [23]. The hybrid method has been proven accurate for Lutetium-177-DOTATATE radionuclide therapy planning [23] as well as for I-131 [24].

This study aimed to assess the accuracy of patient-specific, MC-based absorbed dose calculations for the liver and tumours in I-131-mIBG therapy, based on a hybrid WB planar-SPECT imaging approach. Commercial dosimetry software has historically oversimplified radiation transport, treating tumours in isolation and often ignoring cross-dose effects from neighbouring regions. This approach leads to significant underestimations of absorbed doses, particularly evident in cases involving I-131-mIBG [24]. Recent advancements, however, have introduced innovative solutions utilizing MC methods for radiation dose calculation. By intricately modelling both photon and electron interactions, these methods offer improved accuracy in estimating dose deposition within tumours, adjacent organs, and healthy tissues. Integrating local electron absorption with MC-based photon transport signifies a significant leap in dosimetry software capabilities. This integration allows for a more realistic depiction of dose distribution in complex anatomical environments, thereby improving precision in treatment planning and delivery [25]. Additionally, the aim included an investigation of the absorbed doses obtained when considering both photon and charged particle emissions from I-131, or beta emissions only. The study used voxel-based digital patient phantoms and MC-generated WB and SPECT images. The novel approach of replacing the reconstructed activity distribution with a uniform activity distribution based on the quantified activity was investigated. To the best of our knowledge, this method has not previously been used. Additionally, the rapid advancements in artificial intelligence models for organ segmentation further promotes the clinical implementation of uniform activity distribution for absorbed dose calculations [26]. Dosimetry was performed using imaging of I-123-mIBG with LEHR and ME collimators, and I-131-mIBG imaging with a HE collimator.

## Methods

### Monte Carlo simulations

Three voxel-based digital patient phantoms were created from CT images of three randomly selected retrospective patient SPECT/CT data sets from the Universitas Academic Hospital NM patient database (#1–3). The process of creating these voxel-based digital phantoms has been described by Morphis et al. [27, 28].

**Fig. 1:**
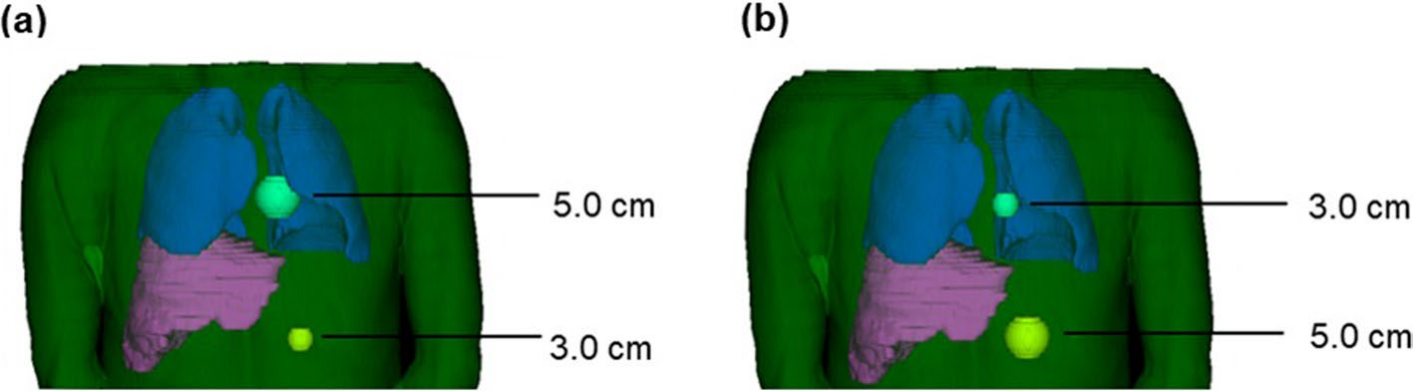
Example of one of the three voxel-based digital patient phantoms showing the lungs, liver and position of the tumours for **a** scenario 1 and **b** scenario 2

Two differently sized spheres (henceforth referred to as tumours) mimicking spherical tumours were included in each of the three digital patient phantoms. Two scenarios for each patient phantom were created (Fig. 1); scenario 1 included a tumour with a diameter of 5.0 cm (65.5 ml) positioned between the lungs and a second tumour with a diameter of 3.0 cm (14.1 ml) positioned below the liver. In scenario 2, the positions of the two tumours were reversed. Relying on clinical experience, the tumour positions were chosen to mimic a NET patient scenario where the contribution of counts from adjacent organs with high activity uptake may impact the accuracy of the tumour activity quantification as well as the tumour average absorbed doses. Additionally, the selection of tumour sizes aimed to represent different impacts of partial volume effects (PVEs), where one having a size of 3.0 cm makes it more susceptible while another with a size of 5.0 cm will be less affected.

The activity concentration [A] in the liver, lungs, tumours, and remainder of the body (all other organs and tissue besides liver, lungs, and tumours—collectively referred to as remainder) was uniformly assigned according to the pharmacokinetic data (biological washout, excluding physical half-life) in Table 1. The [A] values were determined by analysis of clinical diagnostic retrospective mIBG SPECT patient data sets for I-123 and I-131, obtained from the Universitas Academic Hospital NM patient database, with an administered activity (AA) of 370.0 MBq and 185.0 MBq, respectively [29]. Negligible differences were noted between I-123-mIBG and I-131-mIBG biodistributions. The [A] values, representing typical values obtained in clinical scenarios, were uniformly assigned to the organs of all three patients, assuming an identical pharmacokinetic distribution of mIBG across them. Nevertheless, it should be noted that the three patients’ anatomies varied, implying that differences in organ absorbed doses were to be anticipated. The pharmacokinetic data, shown in Table 1, was decay-corrected and normalised to the AA, therefore expressed as kBq/ml/AA. The same pharmacokinetic data was used to simulate WB and SPECT images of the three patient phantoms.

**Table 1:** Decay-corrected pharmacokinetic data determined from mono-exponential fitting of quantified activity values obtained from clinical WB images at different time points p.i

Time (hours p.i)	Decay-corrected activity concentration (kBq/ml) per administered activity ([A]/AA)			
	Liver	Lungs	Tumours	Remainder
1	27.9	15.5	342.3	3.4
4	26.2	14.1	325.2	3.2
6	25.1	13.3	314.2	3.1
24	17.2	7.6	231.0	2.3
36	13.3	5.2	188.2	1.8
48	10.4	3.6	153.3	1.5
72	6.2	1.7	101.7	1.0

The lung and remainder [A]/AA values were included in Table 1 to illustrate the activity distribution models, and not to indicate that lung and remainder doses were calculated. 

The SIMIND MC code [30] was used to model the dual-head Siemens Symbia T16 (Siemens Healthcare, Erlangen, Germany) SPECT/CT system, used at the Department of Nuclear Medicine at Universitas Academic Hospital (Bloemfontein, South Africa), previously validated by Morphis et al. [27, 28]. Anterior and posterior (A/P) WB planar (henceforth referred to as WB) and SPECT projection images were simulated for three radionuclide-collimator combinations, I-123 LEHR, I-123 ME and I-131 HE, as described previously [27, 28].

WB images were simulated for five time points corresponding to 1, 4, 6, 24 and 36 h p.i for I-123-mIBG and 6, 24, 36, 48 and 72 h p.i for I-131-mIBG. The last I-131 time-point was included to represent a typical clinical imaging protocol where the longer physical half-life of I-131 allows for assessment of the washout kinetics. SPECT projection images were simulated for the time point 24 h p.i for both I-123 and I-131. Activity distributions at the different time points were obtained by combining the data in Table 1 with the AA (370.0 MBq and 185.0 MBq) and physical decay constants of I-123 and I-131, respectively.

WB images were simulated (including the addition of Poisson noise) with an image matrix and pixel size of 256 × 1024 and 2.4 × 2.4 mm^2^, respectively. Sixty SPECT projection images with a matrix and pixel size of 128 × 128 and 4.8 × 4.8 mm^2^, were simulated (including the addition of Poisson noise) in step-and-shoot mode using a non-circular orbit-of-rotation estimated from the digital phantom. An energy window of 15% was centred over the 159 keV and 364 keV photopeaks of I-123 and I-131, respectively. A large number of histories were simulated to ensure essentially noise-free MC data [19]. Images were simulated to match acquisition parameters (WB: scan speed of 6.0 cm/min and scan length of 200 cm, SPECT: 40 s/projection), as described previously [27, 28].

### Image processing and activity quantification

**Fig. 2:**
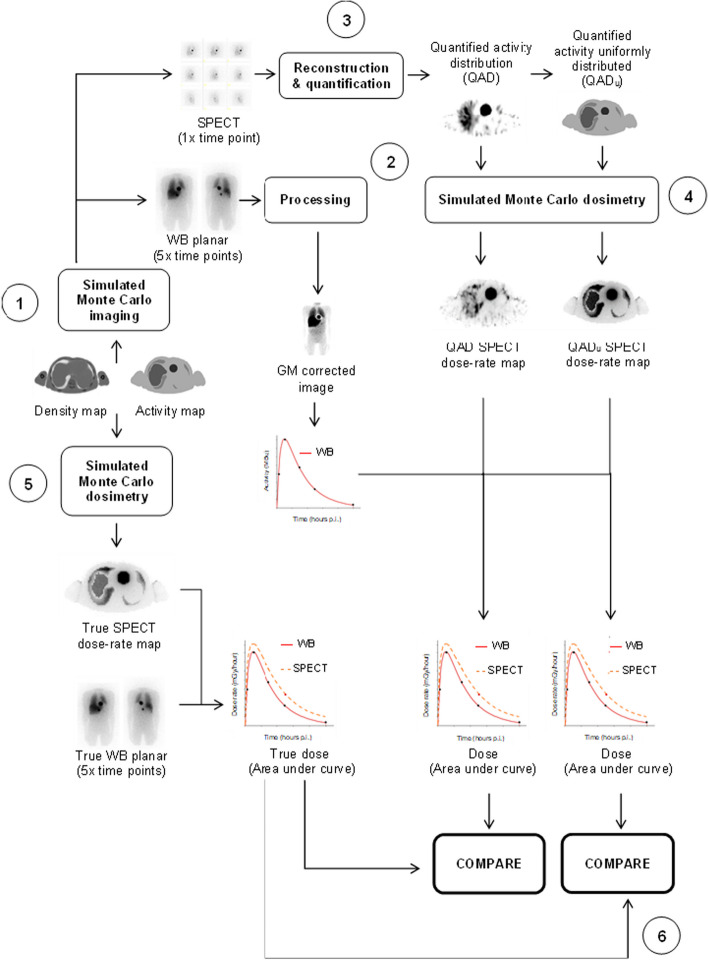
Schematic flowchart of the various steps involved in the assessment of dosimetry accuracy. In step (1) SPECT and WB images are simulated using the density and activity maps as input for the MC simulator. (2) The WB images are processed. (3) The SPECT projections are reconstructed and the activity in each VOI is quantified. (4) MC dosimetry is performed for both QAD and QAD_u_. (5) Voxel-based MC dosimetry is performed on the true activity map to obtain the true dose distribution. (6) The calculated absorbed dose from QAD and QAD_u_ is compared to the true absorbed dose, for the liver, 3.0 cm, and 5.0 cm tumour

The schematic flowchart in Fig. 2 illustrates the various steps involved in the quantification and dosimetry processes.

The simulated WB images (Fig. 2, step 1) were processed with LundADose [31] (Fig. 2, step 2). Scatter correction of individual A/P WB images was performed by Wiener deconvolution filtering with scatter kernels, pre-calculated by MC simulations of a point source at different water depths. These simulations incorporated both scatter in the phantom, backscatter from the compartment behind the crystal, as well as collimator scatter and septal penetration [22]. A geometric mean image was calculated from the scatter-corrected WB images, and attenuation correction was applied by multiplication with a patient-specific map of attenuation correction factors. The attenuation map was created from a projection of the patient CT dataset, scaled to match the attenuation of 159 keV and 364 keV, respectively [22]. These corrected WB images were intentionally not converted to activity data as their sole purpose was to obtain the shape of the time activity curve (TAC) for the respective organs. Despite this, scatter and attenuation corrections were performed ensuring images with improved image contrast for organ and tumour delineation [22]. To limit the contribution of over- and underlying activity from other source organs, regions of interest (ROIs) smaller than the physical size of the source region but large enough to ensure good count statistics, were drawn. Time activity curves (TACs) were determined by fitting mono-exponential functions to the mean image counts within the ROIs covering the liver and the two tumours.

SPECT projection images (Fig. 2, step 3) were reconstructed using OS-EM iterative reconstruction developed by Frey and Tsui [22], including CT-based attenuation correction, model-based scatter correction using the Effective Scatter Source Estimation algorithm, and CDR compensation accounting for both septal penetration and collimator scatter using pre-calculated MC simulated kernels [32]. No postfiltering was performed. Reconstruction was performed with a pre-determined number of OS-EM updates (60 OS-EM updates) [29], resulting in SPECT images with a matrix dimension and voxel size of 128 × 128 × 128 and 4.8 × 4.8 × 4.8 mm^3^, respectively. The reconstructed images were converted to units of activity using a pre-determined calibration factor obtained from a point source simulation in air [29]. Volumes of interest (VOIs) were manually delineated to represent the lungs (representative spherical volume of interest (VOI) large enough to not be influenced by the PVE), liver (physical size), two tumours (physical size) and remainder of the body (representative spherical VOI large enough to not be influenced by the PVE). The activity in each tumour VOI was corrected for PVEs by applying recovery coefficients obtained from pre-determined recovery curves [29]. The recovery coefficients were selected based on tumours of equivalent volume to the delineated VOI. The error in the calculated activity for the different VOIs was defined according to Eq. 1, as the percentage difference between the recovered activity concentration ([*A*]_*recovered*_) and the true activity concentration ([*A*]_*true*_), as defined in the SIMIND digital patient phantom simulation setup,

1 $$Quantification\,error\,\left( \% \right) = \frac{{\left[ A \right]_{{re{\text{cov}} ered}} - \left[ A \right]_{true} }}{{\left[ A \right]_{true} }} \times 100$$

### Dosimetry

Voxel-based dosimetry was performed using full MC radiation transport (Fig. 2, step 4), based on the EGSnrc MC software, and incorporated in LundADose. The MC absorbed dose rate calculations were performed for I-131, based on activity distributions derived from SPECT images combined with density maps obtained from the co-registered CT images. This yielded three-dimensional images with voxel values in units of mGy/hour. When using the quantified I-123 SPECT images as input to the I-131 absorbed dose rate calculation, the activity distribution of I-131 was assumed to be identical to that of I-123, and the obtained mean dose rates in the VOIs were later renormalised (see below). The cut-off energy for I-131 photon and electron emissions was set to 0.01 and 0.1 MeV, respectively. Dosimetry MC calculations were performed with (i) both photons and charged particle emissions, and (ii) beta particle emissions only, using 100 million histories.

Two image sets defining the activity distribution were used as input for the MC absorbed dose rate calculations. These were (i) the quantified activity distribution obtained after SPECT reconstruction (QAD) and (ii) the activity distribution from the SPECT images with the additional step of redistributing the activity within the respective VOI (lungs, liver, tumours, remainder of the body) such that the activity distribution became uniform within the VOI (QAD_u_) (Fig. 2, step 4). The latter (ii) was investigated to mitigate the Gibbs artefacts resulting from CDR compensation [33]. These ring-shaped artefacts are characterised by an increased count level at the edges of an object and a corresponding decreased count level in the object centre [20]. In principle, they may affect the accuracy of the calculated absorbed dose in sub-organ volumes due to the incorrect activity distribution.

For each patient phantom scenario and radionuclide-collimator combination (I-123 LEHR, I-123 ME and I-131 HE), the I-131 absorbed doses to the liver and two tumours were calculated. From the TACs obtained from I-123-mIBG and I-131-mIBG planar images, mono-exponential effective half-lives were determined for each of the three regions. Effective half-lives for I-123-mIBG were recalculated to I-131-mIBG considering the difference in physical half-lives between I-123 and I-131. Mean absorbed dose rate values at 24 h p.i were obtained from the absorbed dose rate images using VOIs for each of the three regions. For the absorbed dose rate images calculated using the I-123 SPECT images as input, the different effective half-lives of I-123 and I-131 were considered by renormalizing the VOI mean absorbed dose rates. The absorbed dose rates at 24 h were combined with the I-131 effective half-lives to obtain the absorbed doses by analytic integration from zero to infinity. This was conducted for the liver and two tumours, in three patient phantoms, each with two scenarios and three camera settings, using both the QAD and QAD_u_ SPECT datasets.

The accuracy of the estimated I-131 mean absorbed doses was assessed by comparison to the true absorbed doses. These were obtained by performing MC absorbed dose calculation directly from the predefined activity distributions assigned to the voxel-based digital patient phantoms (Fig. 2, steps 5 & 6). The absorbed dose accuracy (%) was reported for the liver and both tumours according to Eq. 2,

2 $$Absorbed\,dose\,accuracy\,\left( \% \right) = \frac{{D_{calculated} - D_{true} }}{{D_{true} }} \times 100$$

## Results

### Time-activity curves

**Fig. 3:**
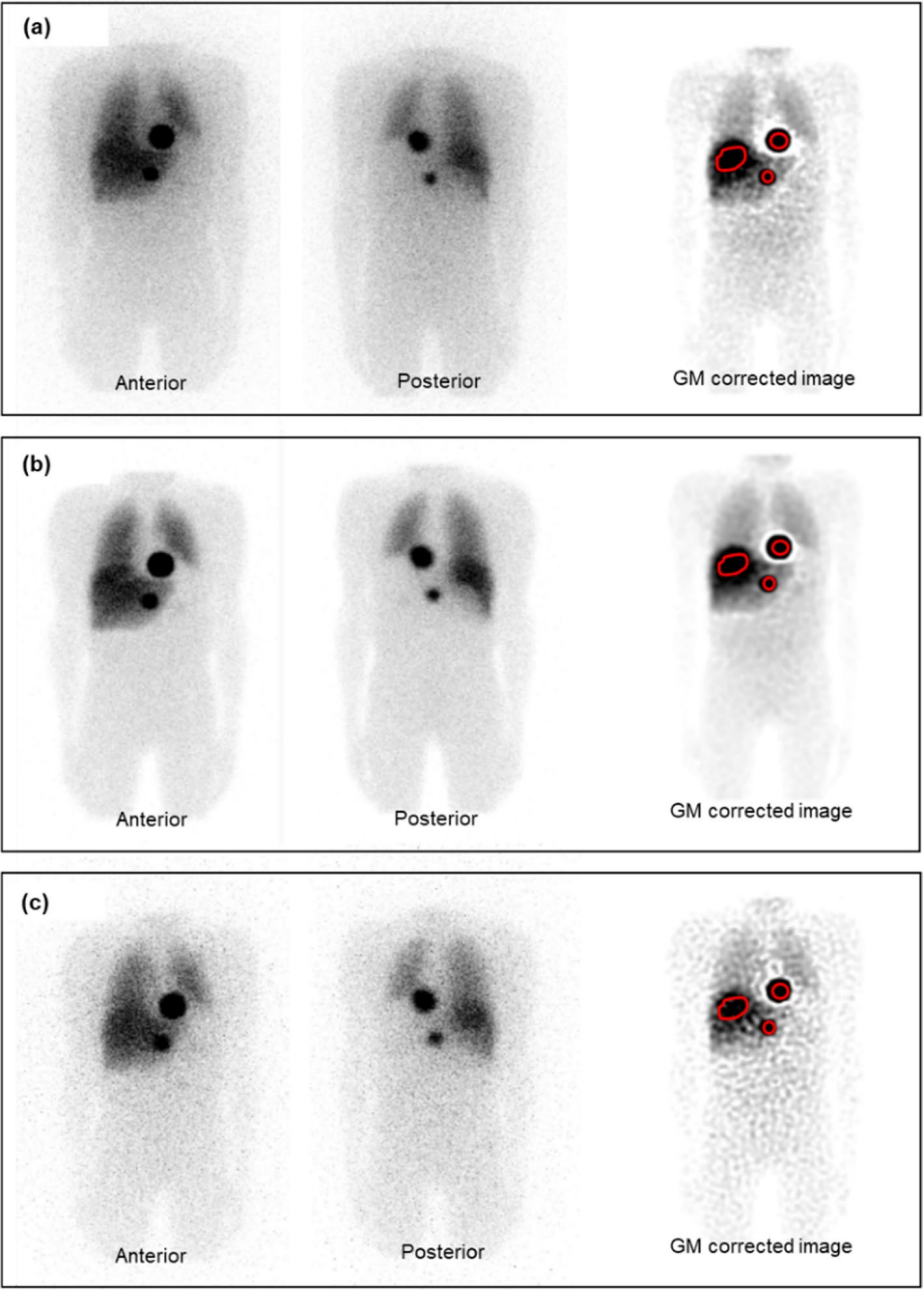
Examples of simulated anterior and posterior WB studies and the corresponding GM attenuation and scatter corrected images for **a** I-123 LEHR, **b** I-123 ME, and **c** I-131 HE

Figure 3 shows an example of the simulated A/P WB images, as well as the GM scatter and attenuation corrected image for the three radionuclide-collimator combinations. The tumour and liver ROIs used to create the TACs are also shown in Fig. 3.

Overall, the WB A/P I-123 ME images show improved image contrast compared to the I-123 LEHR and I-131 HE images. This can be attributed to the collimator septal scatter and penetration, which is more pronounced for I-123 LEHR and I-131 HE compared to I-123 ME. Furthermore, due to the lower AA and poorer I-131 HE system sensitivity [28], the I-131 HE images present with more noise. From Fig. 3 it is evident that the GM scatter and attenuation corrected images show an improvement in image contrast for all three radionuclide-collimator combinations.

**Fig. 4:**
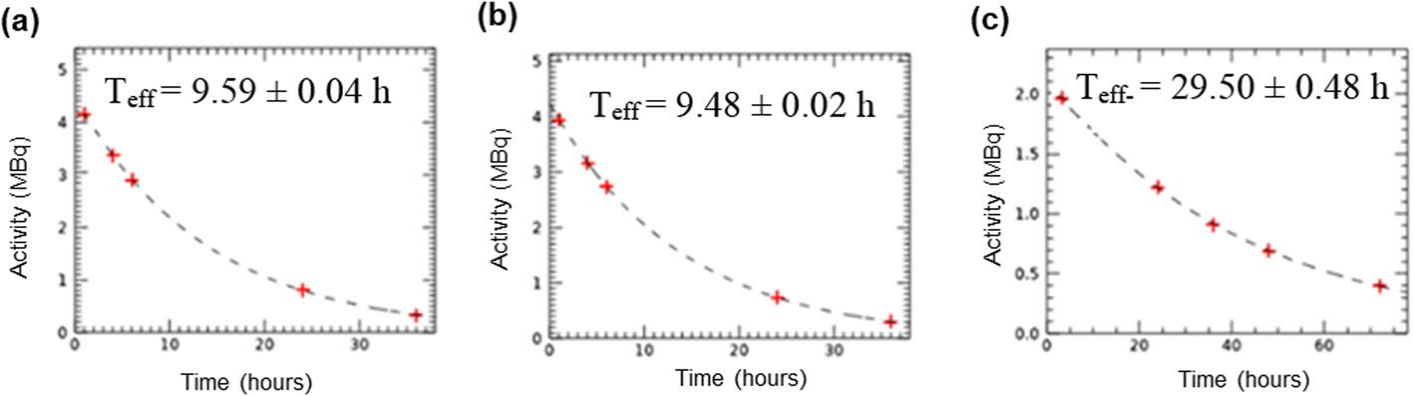
Liver TAC, predicting the shape of the liver time dose rate curve, for **a** I-123 LEHR, **b** I-123 ME and **c** I-131 HE

The fitted liver TACs obtained from the WB images at five different time points, are shown in Fig. 4 for all three radionuclide-collimator combinations.

Overall, there is a good agreement between the liver TACs for I-123 LEHR (T_eff_ = 9.59 ± 0.04 h) and I-123 ME (T_eff_ = 9.48 ± 0.02 h) (Fig. 4a, b). Due to its longer physical half-life, the I-131 HE TAC (Fig. 4c) shows a more prolonged washout from the liver, compared to I-123.

### SPECT activity quantification

The activity quantification errors (Eq. 1), for the liver, 3.0 cm and 5.0 cm tumours, for the three radionuclide-collimator combinations, are shown in Table 2. The results show good agreement between the [*A*]_*recovered*_ and [*A*]_*true*_ for the liver and both tumours in both scenarios for all three patient phantoms.

**Table 2:** Activity quantification error (%) for the liver, 3.0 cm and 5.0 cm tumour, in both scenarios for patient phantoms #1–3 (using Eq. 1)

	Activity quantification error (%)								
	Liver			3.0 cm tumour			5.0 cm tumour		
	I-123 LEHR	I-123 ME	I-131 HE	I-123 LEHR	I-123 ME	I-131 HE	I-123 LEHR	I-123 ME	I-131 HE
Scenario 1, # 1	* − 5.0*	1.9	− 0.8	− 4.1	− 2.0	− 3.8	− 4.1	− 3.1	− 2.6
﻿Scenario 1, # 2	2.7	− 2.1	− 3.1	1.5	3.4	1.2	* − 6.9*	− 6.0	− 5.0
﻿Scenario 1, # 3	4.5	1.9	1.8	0.1	0.0	4.8	− 3.7	− 1.5	− 2.3
Scenario 2, # 1	− 3.5	− 3.8	− 2.1	− 3.0	− 0.9	5.4	0.3	1.6	− 0.5
Scenario 2, # 2	− 2.6	− 0.3	2.1	* − 6.2*	− 5.8	− 1.1	2.2	3.2	2.3
Scenario 2, # 3	3.2	1.1	− 2.4	− 2.8	− 3.4	− 1.1	− 2.0	1.2	0.7
Mean	− 0.1	− 0.2	* − 0.8*	* − 2.4*	− 1.5	0.9	* − 2.4*	− 0.8	− 1.2
SD	4.0	2.3	2.2	2.8	3.1	3.6	3.3	3.4	2.6

The largest quantification errors noted were − 5.0% (#1, scenario 1), − 6.2% (#2, scenario 2), and − 6.9% (#2, scenario 1), for the liver, 3.0 cm and 5.0 cm tumour, respectively. These were observed for all the I-123 LEHR datasets. The average quantification errors (across all radionuclide-collimator combinations) did not exceed − 0.8% for the liver, − 2.4% for the 3.0 cm tumour and − 2.4% for the 5.0 cm tumour.

### Dosimetry

**Fig. 5:**
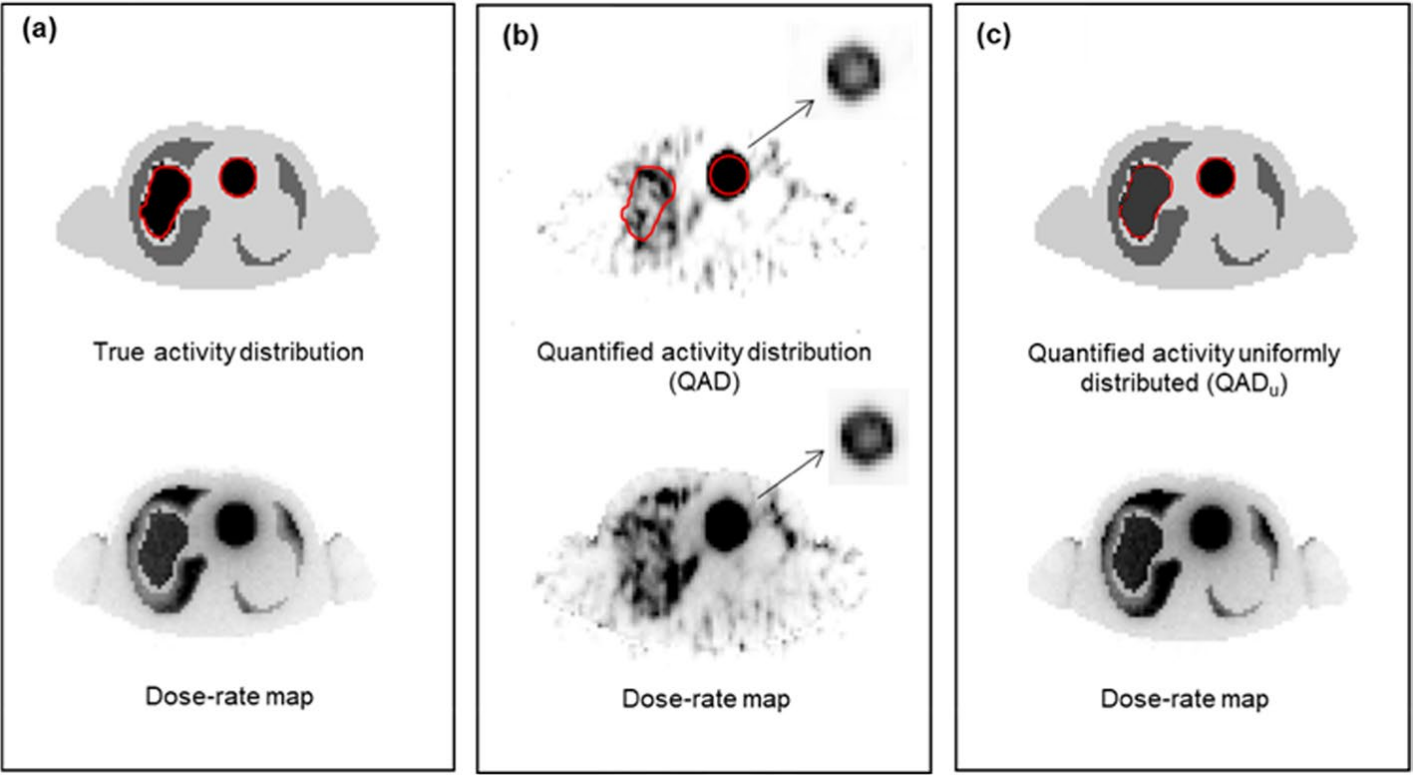
Transverse slices through I-123 LEHR activity maps and corresponding I-131 dose rate maps (photon and charged particle emissions), for **a** true activity distribution, **b** QAD, and **c** QAD_u_. The inserted image of the tumour in Fig. 5b shows the presence of the Gibbs artefact, post-reconstruction, when viewed at an adjusted contrast level. Clinically, this could be misinterpreted as tumour necrosis

Transverse slices through the I-123 LEHR SPECT activity distribution and the corresponding I-131 dose rate maps (both photon and charged particle emissions) are shown in Fig. 5, for (a) the true activity distribution, (b) QAD, and (c) QAD_u_. The liver and 5 cm tumour VOIs, as delineated on the CT images, are also shown.

Figure 5a shows the true dose rate map derived from the true uniform activity distribution at 24 h p.i. Image degrading effects (non-uniform activity distribution and Gibbs artefacts) are visible in Fig. 5b. Due to the practical constraints of the permitted administered radiation activity to patients and the inherent properties of the gamma camera, the quantitative SPECT images are noisy. These noisy activity distribution images result in equally noisy dose rate maps, which do not compare well to the true dose rate map in Fig. 5a. Using the quantified activity obtained from Fig. 5b (QAD) to generate a uniform activity distribution in each VOI (Fig. 5c (QAD_u_)), a dose rate map that visually compares well with the true dose rate map is obtained.

**Fig. 6:**
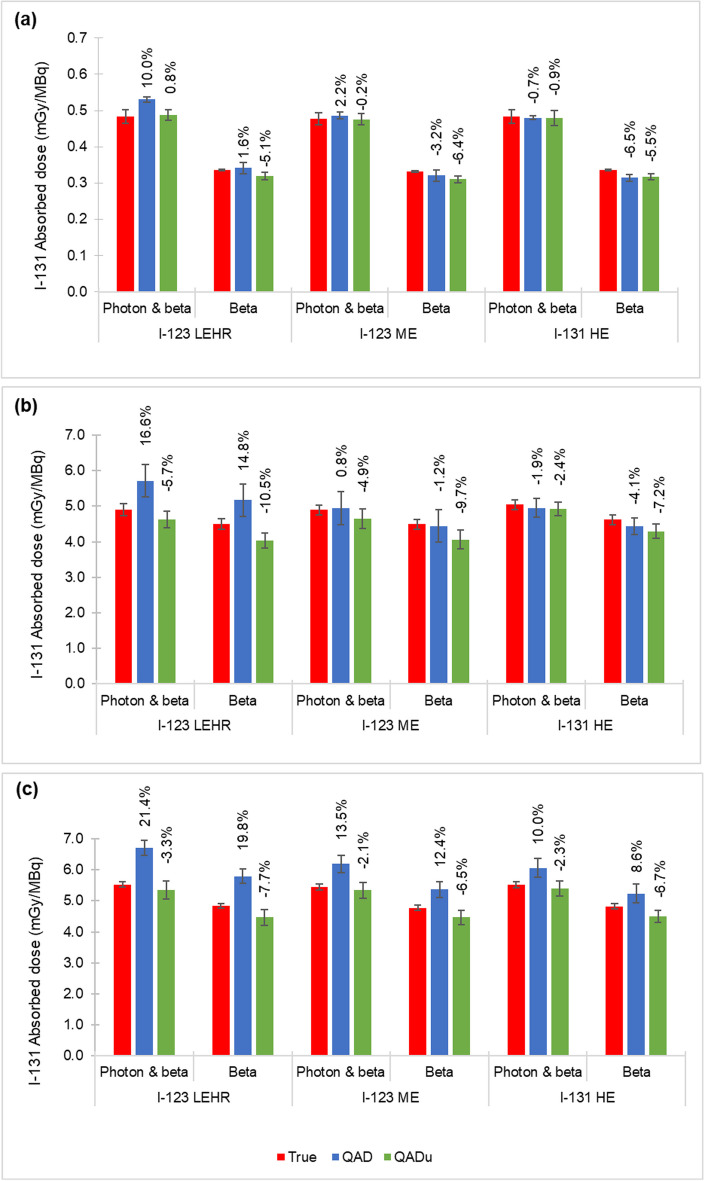
I-131 absorbed dose values calculated from the true activity distribution and QAD and QAD_u_, for the **a** liver, **b** 3.0 cm tumour, and **c** 5.0 cm tumour, averaged over the three patient phantoms and both scenarios, for all radionuclide-collimator combinations. Absorbed dose values reported for photon and charged particle emissions as well as beta only emissions. The values above the QAD and QAD_u_ bars show the percentage difference with respect to the true absorbed dose. The error bars represent one standard deviation of the average absorbed dose values

Figure 6 shows the I-131 absorbed dose values, averaged over all three patient phantoms and both scenarios, with contributions from photon and charged particle emissions as well as beta emissions only, for the liver, 3.0 cm, and 5.0 cm tumours, calculated from the I-123 LEHR, I-123 ME and I-131 HE activity distributions. The values above the QAD and QAD_u_ bars show the percentage difference with respect to the true absorbed dose.

Average percentage differences between true and QAD_u_ calculated absorbed doses were < 9.7% (3.0 cm tumour) and < 7.2% (3.0 cm tumour) for I-123 ME and I-131 HE, respectively. Slightly larger percentage differences between the true and QAD calculated absorbed doses were noted for both I-123 ME (13.5% for the 5.0 cm tumour) and I-131 HE (10.0% for 5.0 cm tumour). For I-123 LEHR, the absorbed doses obtained from QAD_u_ compared well to the true absorbed dose, with percentage differences ≤ 10.5% (3.0 cm tumour). For QAD, overestimated true absorbed doses were obtained, with percentage differences up to 21.4% (5.0 cm tumour). This overestimation can be attributed to the more pronounced effects of septal scatter and penetration for I-123 LEHR contributing to the absorbed dose, despite correction thereof.

The results shown in Fig. 6a show that the liver absorbed dose is predominantly due to beta contribution, with photons contributing 34.8% and 34.3% of the total absorbed dose for QAD and QAD_u_, respectively. In comparison, the absorbed dose from photon contribution is much lower for the 3.0 cm (9.9% and 12.8%) and 5.0 cm (13.5% and 16.5%) tumours (Figs. 6b, 7c). Considering its relatively large size, one can expect more photon interactions within the liver volume. Conversely, most photons originating from the 3.0 cm and 5.0 cm tumours are more likely to escape the tumour volume before depositing their energy.

The results in Fig. 6 are summarised in Table 3, which shows the average absorbed dose values, across all radionuclide-collimator combinations and patient phantom scenarios, for the liver, 3.0 cm, and 5.0 cm tumours. Similar liver absorbed dose values were obtained between the QAD and QAD_u_ image sets. The relatively small standard deviations highlight the small differences in absorbed dose values across all three radionuclide-collimator combinations.

**Table 3:** Average absorbed dose values across all radionuclide-collimator combinations and patient phantom scenarios for the liver, 3.0 cm and 5.0 cm tumour

	*Absorbed dose (mGy/MBq)			
	Photons and beta		Beta	
	QAD	QAD_u_	QAD	QAD_u_
Liver	0.5 ± 0.02	0.5 ± 0.01	0.3 ± 0.02	0.3 ± 0.00
3.0 cm tumour	5.2 ± 0.54	4.7 ± 0.16	4.7 ± 0.51	4.1 ± 0.14
5.0 cm tumour	6.3 ± 0.39	5.4 ± 0.03	5.5 ± 0.35	4.5 ± 0.02

^*^Mean ± one standard deviation

## Discussion

The assessment of radiopharmaceutical therapy planning requires precise dosimetry. Ljungberg et al. [32] used voxel-based digital phantoms, in conjunction with MC simulations, to determine possible sources of error in the quantification process, allowing for the evaluation of absorbed dose distributions. This study provides a similar evaluation procedure for determining the accuracy of the absorbed dose to the liver and tumours in I-131-mIBG therapy, based on clinically realistic voxel-based digital patient phantoms.

The results showed that QAD overestimated the absorbed doses up to 21.4% (5.0 cm tumour I-123 LEHR). Although a recovery coefficient was used to compensates for lost counts in the VOI due to spill-out counts, these spilled-out counts are not removed from the image and ultimately still contribute to the absorbed dose calculated for the tumour VOI. A study by Dewaraja et al. [19] showed that when I-131 SPECT activity was underestimated, the I-131 absorbed dose was also underestimated, but to a lesser extent, and stated that this could be attributed to the spilled-out counts that did not contribute to the VOI activity but did contribute to the absorbed dose.

As highlighted by prior research findings and reaffirmed in our study, a drawback of applying a CDR correction is the potential introduction of Gibbs ringing artefacts [34]. During the validation of a SIMIND Monte Carlo modelled gamma camera for imaging with I-123 and I-131 [26], significant Gibbs artefacts were observed in the reconstructed images of a 4.2 cm diameter sphere when incorporating CDR in the iterative reconstruction process. Given that the sources utilized in this study were 3.0 cm and 5.0 cm in diameter, we anticipated similar Gibbs artefacts. These artefacts can result in an overestimation of the absorbed dose, particularly in smaller objects. Zeng [34] demonstrated that separating image reconstruction from point spread function compensation is an effective method for mitigating Gibbs artefacts. Future research will explore these limitations, building upon the current study.

To address the issues associated with spill out and Gibbs artefacts, the reconstructed activity distribution was replaced with a uniform distribution of the quantified activity (QAD_u_) within the respective VOI, improving dosimetry accuracies by approximately10.5%. Overall, absorbed dose values obtained with QAD_u_ showed improved accuracy. The QAD_u_ approach was based on the rationale that when the real underlying distribution cannot be reliably estimated from the reconstructed SPECT images and therefore remains unknown, the most basic assumption is a uniform activity distribution. This assumption is also intrinsic in the application of recovery coefficients, commonly determined based on objects with uniform activity distribution. The assumption is justified for I-131-mIBG in the liver, as the activity uptake has been observed to be relatively uniform for average liver volumes [35]. A shortcoming of this assumption is that larger tumours may exhibit non-uniform uptake patterns, potentially due to the presence of a necrotic centre. Unlike research-oriented software such as 3D Slicer [26] and ImageJ [36], MIM SurePlan™ is a commercially available software specifically tailored for clinical applications, including image segmentation and dose calculation capabilities [37].

It is important to note that assigning uniform activity distributions to all patient phantom structures during the simulation can be considered a limitation. The question arises as to how accurate organ and tumour absorbed dose values can be obtained from the QAD_u_ approach in cases with a non-uniform initial activity distribution. Future research can explore these scenarios, broadening the scope of our study and potentially enhancing the practical implementation of the QAD_u_ approach in diverse clinical settings.

A wide range of I-131-mIBG liver absorbed dose values have been reported in literature, with values ranging from 0.3 to 1.1 mGy/MBq [1, 9, 38–40] and others ranging from 2.0 to 5.5 mGy/MBq [19, 32, 41, 42]. The absorbed dose values (photon and charged particle emissions) of 0.5 ± 0.02 mGy/MBq (for both QAD and QAD_u_) obtained in this study fall well within these reported values. It is important to note that the absorbed doses for beta emissions only resulted in values up to 30.0% lower than when photon emissions were also considered.

I-131-mIBG tumour absorbed doses ranging between 0.2 and 17.0 mGy/MBq have been reported in literature [19, 38, 39]. Tumour absorbed dose values (photon and charged particle emissions) reported in Fig. 6 vary between 4.6 mGy/MBq and 6.7 mGy/MBq. When only beta emissions were considered, absorbed doses were up to 12.5% lower. This indicates that the assumption made by some commercial dosimetry software programs, which treat tumours as isolated objects, not including cross-dose to and from other source and target organs, may result in significant dose underestimations of I-131-mIBG studies. Similar findings were reported by Grimes et al. [43].

Roth et al. [23] demonstrated the suitability of the hybrid method in planning Lutetium-177-DOTATATE radionuclide therapy. Our study has shown its suitability for I-131-mIBG radionuclide therapy planning. By accounting for the three key factors, prior to engaging in the hybrid WB planar-SPECT/CT quantification of patients, we propose that this method holds promise for application in a broader spectrum of treatments.

Our proposed technique holds promise for I-131-mIBG dosimetry in treatment planning, however, transitioning from planning to verification poses several challenges (e.g. gamma camera deadtime, patient-specific factors including patient motion and image quality) that require careful consideration. Future research endeavours should concentrate on addressing these challenges comprehensively. This could involve integrating advanced imaging modalities, refining deadtime correction algorithms, and enhancing patient-specific dosimetric modelling techniques. By addressing these aspects, we can further enhance the accuracy and reliability of our dosimetric approach.

## Conclusions

The hybrid planar-SPECT/CT method for dosimetry has proven effective for personalised treatment planning of I-131 radiopharmaceutical therapy with either I-123 or I-131 imaging. Accurate dosimetry can be obtained for the liver and tumours, with diameters as small as 3.0 cm. The quantification accuracy obtained with I-123 LEHR and I-131 HE is acceptable despite the poorer image quality.

The novel approach of replacing the non-uniform reconstructed activity distribution with a uniform activity distribution based on the quantified activity, results in a calculated absorbed dose resembling the true absorbed dose value. This is an acceptable approach when applying partial volume correction using the standard recovery coefficient method, to mitigate the Gibbs artefacts resulting from CDR compensation in SPECT images.

## Data Availability

The datasets generated during the study are available from the corresponding author on reasonable request.

## Abbreviations

[A]: Activity concentration
A/P: Anterior/posterior
AA: Administered activity
CDR: Collimator–detector response
CT: Computed tomography
GM: Geometric mean
HE: High energy
I-123: Iodine-123
I-131: Iodine-131
LEHR: Low energy high resolution
MC: Monte Carlo
ME: Medium energy
mIBG: Metaiodobenzylguanidine
NETs: Neuroendocrine tumours
NM: Nuclear medicine
OS-EM: Ordered subset expectation maximization
p.i: Post injection
QAD: Quantified activity distribution
QAD_u_: Quantified activity distribution uniform
ROI: Region of interest
ROIs: Regions of interest
SPECT: Single photon emission computed tomography
TAC: Time activity curve
TACs: Time activity curves
T_eff_: Effective half-life
VOI: Volume of interest
VOIs: Volumes of interest
WB: Whole body
